# Assessment of dynamic knee angle deviations in the frontal plane in physiotherapy clinical practice: intra- and inter-rater reliability of an application and agreement with two AI-models

**DOI:** 10.1186/s12891-026-09958-9

**Published:** 2026-05-12

**Authors:** Lukas Janisch, Lilo-Marie Maack, Dominik Fohrmann, Carlos J. Marques

**Affiliations:** 1 Department Performance, Neuroscience, Therapy and Health, Faculty of Health Sciences, MSH Medical School Hamburg, Program Bachelor of Science Physiotherapy, Am Kaiserkai 1, Hamburg, D-20457 Germany; 2 Faculty of Medicine, Medical School Hamburg, Hamburg, Germany; 3 Institute of Interdisciplinary Exercise Science and Sports Medicine, MSH Medical School Hamburg, Am Kaiserkai 1, Hamburg, D-20457 Germany

**Keywords:** Functional Assessments, AI-Based Human Pose Estimation, Prehab, Knee kinetics, Rehabilitation, Anterior cruciate ligament (ACL) injury, Injury prevention

## Abstract

**Background:**

Functional leg axis deviations, particularly dynamic knee valgus (DKV), are considered key risk factors for lower-extremity injuries. In physiotherapy practice there are no standardized tools to quantitatively assess functional leg axis deviations within a treatment session. While application-based 2D analyses offer accessible options, the agreement between manual and automated, AI-based Human Pose Estimation (HPE) methods remains underexplored. Here, the aims were to investigate the intra- and inter-rater reliability of a manual application-based method (PhysioMaster®); to quantify the agreement between two AI-based HPE models (OpenPose, BlazePose); and to explore which functional test, Single-Leg Squat (SLS), Single-Leg Hop for Distance (SLH), or Single-Leg Landing (SLL), elicits the most pronounced leg axis deviations.

**Methods:**

Sixteen healthy adults (8 females) performed three standardized single-leg tasks (SLS, SLH, SLL) with each leg. Knee angles in the frontal plane were measured from monocular video images using the PhysioMaster® application (two raters × two ratings) and compared with automated 2D analyses using OpenPose and BlazePose. Reliability and agreement were evaluated using Intraclass Correlation Coefficients, Lin’s Concordance Correlation Coefficient (CCC), and Bland–Altman analyses. Linear Mixed Model (LMM) analyses were carried out to explore which functional task would be the most effective.

**Results:**

The PhysioMaster® application showed excellent intra- and inter-rater reliability across all tests (ICC ≥ 0.95). OpenPose demonstrated excellent agreement with manual application-based measurements (CCC = 0.95; Bias = 0.87°; LoA = –4.32° to 6.05°), while BlazePose achieved good agreement (CCC = 0.89; Bias = –0.67°; LoA = –8.76° to 7.42°). LMM analyses revealed that all three tasks significantly evoked knee angle deviations. The SLL evoked the largest valgus angles (Estimate = –8.29°, *p* < .001), while the SLS elicited the highest varus angles (Estimate = 7.92°, *p* < .001).

**Conclusion:**

Both, manual application-based and AI-based 2D methods, provided reliable and largely consistent assessments of knee valgus angles in the frontal plane during functional testing. OpenPose achieved the best agreement with manual application-based reference values, and the SLL was the most suitable task for detecting DKV. While the results support the integration of manual application-based and AI-assisted posture analysis into clinical physiotherapy, further research on concurrent validity of all three methods used against 3D motion capture is needed.

**Supplementary Information:**

The online version contains supplementary material available at 10.1186/s12891-026-09958-9.

## Background

Functional leg axis deviations, particularly dynamic knee valgus (DKV), are considered major biomechanical risk factors for injuries of the lower extremity, including anterior cruciate ligament ruptures and patellofemoral pain syndrome [1–3]. These deviations represent complex, multi-planar movement patterns characterized by hip adduction and internal rotation, knee abduction, and foot pronation [3–5]. In physiotherapy practice, the accurate assessment of such deviations is essential for identifying modifiable risk factors, to guide individualized training programs, and to evaluate therapeutic progress [6, 7].

Traditionally, physiotherapists have relied on visual inspection to assess lower limb alignment during functional tasks. While clinical experience enables approximate judgments, these assessments are inherently subjective and lack quantitative precision [8–10]. To strengthen evidence-based decision-making, objective and reproducible measurement tools are required. These should translate functional observations into reliable numerical data [3].

Three-dimensional (3D) motion analysis is widely regarded as the gold standard for quantifying movement kinematics [11, 12]. However, such systems are costly, their use is time-intensive, and they require well-trained personnel, limiting their clinical feasibility [13]. As a result, two-dimensional (2D) video-based approaches have gained increasing attention as practical and low-cost alternatives. Previous studies have shown that the validity of 2D video-based approaches, when compared with 3D motion analysis systems, varies and is generally low to moderate for frontal plane angle measurements. Accordingly, current evidence does not support the use of 2D video analysis for assessing lower extremity frontal plane kinematics when high measurement accuracy is required [14–16]. Nevertheless, three-dimensional (3D) motion analysis systems are not easily integrated into routine clinical physiotherapy practice due to the aforementioned limitations. In contrast, two-dimensional (2D) video-based systems offer a quantitative approach that presents several advantages compared with commonly used visual inspection methods. Among them, mobile applications such as the PhysioMaster^®^ application allow users to manually define anatomical landmarks on still images to calculate planar joint angles. Several studies have demonstrated high intra- and inter-rater reliability for 2D video-based methods in evaluating the frontal plane projection angle (FPPA), a common measure of knee alignment in the frontal plane [17–20].

At the same time, the rapid progress of computer vision, sometimes also referred to as computer image recognition, and artificial intelligence (AI) have enabled the development of Human Pose Estimation (HPE) models, such as OpenPose and BlazePose. These algorithms can automatically detect anatomical landmarks on images or video frames using deep learning [21, 22]. These models offer fully automated markerless joint detection, potentially allowing for standardized, rapid, and objective movement analysis in clinical and field settings [13, 23]. However, despite growing use in sports science, their integration into physiotherapy research remains limited [24].

Existing literature suggests that 2D application-based measurements can yield good to excellent reliability [11, 17, 18, 20] while HPE systems demonstrate promising accuracy in static and simple dynamic movements [25, 26]. Nonetheless, agreement between automated and manual 2D methods under real-world conditions, particularly during complex, single-leg tasks, has not been systematically evaluated [27, 28]. Furthermore, it remains unclear which functional task provides the most distinct and reproducible representation of leg axis deviations in healthy adults [3, 4].

In order to integrate manual application- or HPE-based FPPA assessments into physiotherapy clinical practice, key metrics such as reliability, concurrent validity, and practicality must be investigated. The present study focused on assessing two components of reliability (intra- and inter-rater reliability) of the manual application-based method, as well as the agreement between the manual application-based method and two HPE methods.

In this study data acquired during the performance of three single-leg functional tests was used to compare manual application-based and AI-based measurement approaches. The study pursued three aims. First, to examine the intra- and inter-rater reliability of the PhysioMaster^®^ application for measuring knee valgus and varus angles in the frontal plane. Second, to assess the agreement between two AI-based HPE models, OpenPose and BlazePose, and the manual application-based measurements. Third, to analyze which functional test produces the greatest valgus or varus deviations of the lower limb. The tests included the Single-Leg Squat (SLS), the Single-Leg Hop for Distance (SLH), and the Single-Leg Landing (SLL). Accordingly, the following study hypotheses were formulated: (H1) The intra- and interrater reliability of the manual application-based method (PhysioMaster^®^) for assessing knee varus and valgus angles is good to excellent; (H2) the agreement between the manual application-based method and the two HPE methods is good; and (H3) one of the three functional test is the most appropriate for eliciting functional knee varus or valgus deviations.

## Materials and methods

### Study design

This study was designed as a cross-sectional, method comparison and reliability study. The report follows the Strengthening the Reporting of Observational Studies in Epidemiology (STROBE) guideline for observational research [29] and the Guideline for Reporting Reliability and Agreement Studies (GRRAS) [30].

The study was conducted at the motion analysis laboratory of the MSH Medical School Hamburg between February and March 2025. Ethical approval was obtained from the institutional ethics committee (MSH-2024/372). All participants provided written informed consent prior to data collection. The procedures adhered to the Declaration of Helsinki (1964) and its later amendments.

### Sample

A convenience sample of 16 healthy adults (8 female, 50%) was recruited through local networks. Inclusion criteria were: age ≥ 18 years; absence of acute musculoskeletal injuries of the lower extremities; and ability to perform all functional tests. Exclusion criteria included acute pain; recent surgery; pregnancy; or conditions limiting load-bearing activities. The sample size was determined pragmatically based on feasibility and in accordance to previous pilot studies on 2D motion analysis [11, 19].

### Procedures

At the beginning, participant demographics (age, sex, body height, body mass, BMI) were recorded. Body mass was measured with the use of a weighing scale (803, Seca GmbH and Co KG, Hamburg, Germany). Body height was assessed with the use of a stadiometer (213, Seca GmbH and Co KG, Hamburg, Germany). Ahead of performing the functional tests, each participant performed a warm-up of 5 min running on a treadmill at a self-selected moderate pace. Afterwards, a static frontal plane picture of each subject was recorded. Ahead of the test trials the participants performed three practice trials with each leg in each of the single-leg functional tests. Subsequently, each participant completed three standardized single-leg functional tasks with each leg, namely: Single-Leg Squat (SLS); Single-Leg Hop for Distance (SLH); Single-Leg Landing (SLL). A detailed description of the standardized instructions used during functional testing and criteria for invalid attempts are provided in the appendix Table A1. The test order was randomized and the three consecutive trials on each side were performed. These functional tests were widely used in the past to evaluate lower-limb alignment and neuromuscular control [3, 4]. All tests were performed barefoot on an even surface. Three valid trials were collected for each leg and task. A trial was considered valid, if the participant sustained balance, did not use the contralateral leg for support, and landed or squatted in a controlled manner for at least 3 s.

### Video acquisition

All movements were recorded using an iPad (8th generation, 10.2”, 120 Hz, Apple Inc., Cupertino, CA, USA) positioned 2.5 m in front of the participant on a tripod with the camera’s lens positioned at height of the greater trochanter. Recordings were taken in slow-motion mode (720 p, 120 fps) under consistent lighting conditions. Participants received standardized verbal instructions and practiced each test before recording.

### Data processing

From each valid trial, one still frame was extracted for analysis. The selected frame corresponded to the moment when the hip joint reached its lowest vertical position in the frontal-plane video. This time point was operationally defined as the presumed instant of maximum knee flexion, because no sagittal-plane recordings were available. For protection and data privacy reasons all images (extracted frames) were recorded without the head of the participant and were assigned with coded IDs before further processing. A total of 320 images ([16 participants × 3 tasks × 3 repetitions × 2 sides] + [16 participants x 2 static pictures] were analyzed after quality screening.

### Application-based manual knee angle measurement

**Fig. 1:**
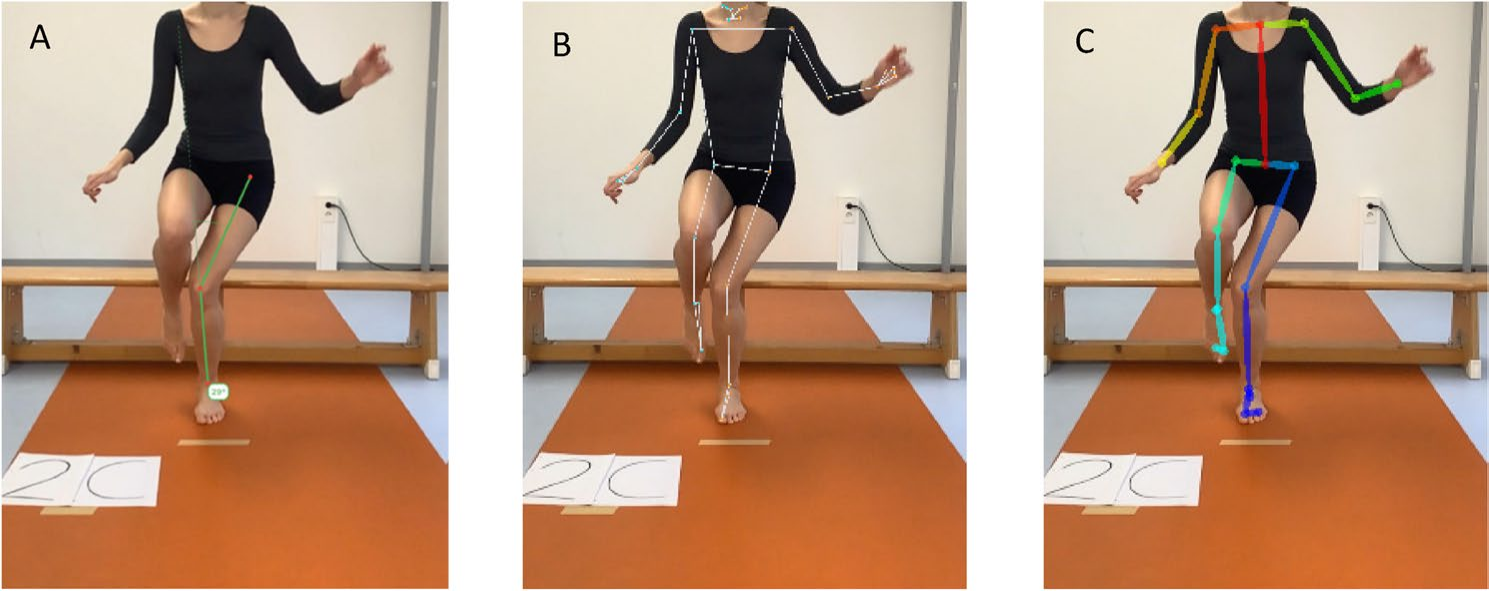
Example of the measurement of the frontal plane projection angle (FPPA) on the same image with: (**A**) the manual application-based method (PhysioMaster®) and the two Human Pose Estimation (HPE) AI-methods, (**B**) BlazePose and (**C**) OpenPose

Application-based manual measurement of the frontal plane knee angle was performed with the use of the PhysioMaster^®^ application (Version 2.2.5 + 41, TrinusLab d.o.o., Omisalj, Croatia) on the same iPad. The frontal plane projection angle (FPPA) was calculated by marking the following three anatomical landmarks: the estimated hip joint center, the center of the patella, and the ankle joint center. The hip joint center was estimated to be medial and inferior to the Anterior Superior Iliac Spine and medial and superior to the greater trochanter. The center of the patella was defined as the midpoint of the patella at patella height, and the ankle joint center was defined as the midpoint between the medial and lateral malleoli. The knee angle was defined as the internal angle between the segments hip–knee and knee–ankle. Negative values represented knee valgus deviations; positive values represented knee varus deviations (Fig. 1a).

Two licensed physiotherapists (LJ, LMM) independently performed all FPPA-angle measurements on all images with the use of the application at two time points. A time window of at least three weeks between the two readings was set to minimize recall bias. The raters were blinded to each other’s measurements and to the results from the AI-based analyses. Each rater entered all measurements manually into a spreadsheet for further processing.

### AI-based automated knee angle measurement

The automated analysis was conducted using the following two open-source HPE models: OpenPose [22], a multi-person model that can detect up to 135 landmarks; and BlazePose [21], a model optimized for mobile and real-time applications with up to 33 landmarks. Both models were implemented via custom Python scripts (version 3.13, python.org) and applied to the same extracted still images as were used during the application-based manual annotation. For each model, the coordinates of the hip, knee, and ankle landmarks were identified automatically. The FPPA was calculated from the two-dimensional landmark coordinates as the angle between the vectors from hip to knee and from knee to ankle landmark. Figure 1B-C shows the detection of the anatomical landmarks of both HPE models used. Outputs were exported as numeric data for statistical analyses. For both AI-based models, all landmark coordinates were exported before data screening. To identify potential detection errors, FPPA values were first inspected for statistical outliers based on the distribution of the complete dataset. Values outside the interval of mean ± two standard deviations were visually checked. Exclusion was applied only when a clear detection error was present, such as missing landmarks, misaligned joints or anatomically implausible segment orientations.

All image data were pseudonymized and stored locally on an encrypted institutional server. No identifiable information was shared or uploaded externally. Data processing and analyses were conducted in compliance with the European General Data Protection Regulation (GDPR, 2016/679).

### Statistical analysis

Descriptive statistics (mean ± SD, minimum, maximum, median) were computed to characterize the sample and to present the data for all measurement methods used.

To address the first research question on intra- and inter-rater reliability, Intraclass Correlation Coefficients (ICC) were calculated [31]. A two-way mixed-effects model (ICC 3,1) was selected because it assesses measurement consistency between two fixed raters on single measurements [32].

To address the second research question on the agreement between manual Application-based and AI-based methods, three complementary approaches were used. First, ICC (3,1) was calculated to quantify consistency between methods. Second, Lin’s Concordance Correlation Coefficient (CCC) [33] was computed using the DescTools package in R, capturing both precision (Pearson’s r) and bias correction (Cb). Third, Bland–Altman analysis [34] was performed to visualize systematic bias and to determine the 95% limits of agreement (LoA = Bias ± 1.96 × SD). Normality of residuals was checked using QQ-plots.

According to Koo & Li (2016) [32], ICC and CCC values ≤ 0.50, between 0.50 and 0.75, between 0.75 and 0.90 and ≥ 0.90, should be respectively interpreted as poor, moderate, good or excellent reliability or agreement.

The third question, whether there is a functional test that best evokes functional valgus or varus leg axis deviations was investigated with two linear mixed models (LMMs). In both models the dependent variable was “knee angle”. In the first model, only valgus (negative FPPA angles) or neutral (0°) measurement results were included. In the second model, only varus (positive FPPA angles) and neutral (0°) measurement results were included. In both models, the fixed effects variables included were “functional task” (Static, SLS, SLH, SLL), measurement time (first and second reading); rater (rater 1 and 2) and sex (male, female). A random intercept per participant was set in each model. In both models, the model terms were tested with the Satterthwaite approximation and Type III sum of squares was used. The models were fitted using the restricted maximum likelihood estimator. Pairwise comparison of the estimated marginal means for each functional test were conducted using the Bonferroni adjustment of the alpha to find the most effective test to elicit functional valgus or varus deviations.

All statistical tests were carried out with JASP version 0.95.4 (University of Amsterdam) and RStudio 2023.06.0 (RStudio Team, Boston, MA). The significance level was set at 0.05 throughout.

## Results

### Participant characteristics

A total of 16 participants (8 female) completed all measurements. The mean age was 31.8 ± 8.9 years, mean height 176.4 ± 9.6 cm, and mean body mass 75.0 ± 11.3 kg (BMI = 24.0 ± 2.2 kg/m²). No adverse events or dropouts occurred during data collection. Demographic characteristics are summarized in Table 1.

**Table 1:** Demographic data of the sample

Variables	All	Female	Male
n	16	8	8
Age (Years)	31.8 ± 9.2	30.3 ± 7.0	33.3 ± 11.2
Body height (cm)	176.4 ± 9.8	168.8 ± 6.9	183.9 ± 5.3
Body mass (kg)	75.0 ± 11.7	67.3 ± 8.7	82.8 ± 9.0
BMI (kg/m^2^)	24.0 ± 2.3	23.6 ± 2.8	24.4 ± 1.7

Values are Mean ± SD

*BMI* Body Mass Index

### Intra- and inter-rater reliability of the application-based manual FPPA angle measurement

The manual measurement with the use of the PhysioMaster^®^ application showed excellent intra- and inter-rater reliability across all three functional tasks (SLS, SLH, SLL). Intra-rater reliability ranged from ICC = 0.95 to 0.97 for both raters (Table 2), while inter-rater reliability reached ICC values of 0.96 (95% CI 0.95–0.97) (Table 3).

**Table 2:** Intra-Rater Reliability for rater 1 and 2

Test	*n*	Rater 1		Rater 2	
		ICC	95% C.I.	ICC	95% C.I.
Static	32	0.81	0.65–0.90	0.65	0.39–0.81
SLS	96	0.98	0.96–0.98	0.97	0.95–0.98
SLH	96	0.97	0.96–0.98	0.97	0.95–0.98
SLL	96	0.98	0.97–0.99	0.97	0.96–0.98

ICC 3,1 type; *n* = number of frames use in each analysis

*SLS* Single-leg squat, *SLH* Single-leg hop for distance, *SLL* Single-leg landing

**Table 3:** Inter-Rater Reliability

Test	*n*	1st Reading		2nd Reading	
		ICC	95% C.I.	ICC	95% C.I.
Static	32	0.62	0.35–0.80	0.63	0.37–0.80
SLS	96	0.94	0.94–0.96	0.96	0.94–0.97
SLH	96	0.96	0.94–0.97	0.96	0.94–0.97
SLL	96	0.97	0.96–0.98	0.97	0.96–0.98

ICC 3,1 type; *n* = number of frames use in each analysis

*SLS* Single-leg squat, *SLH* Single-leg hop for distance, *SLL* Single-leg landing

**Fig. 2:**
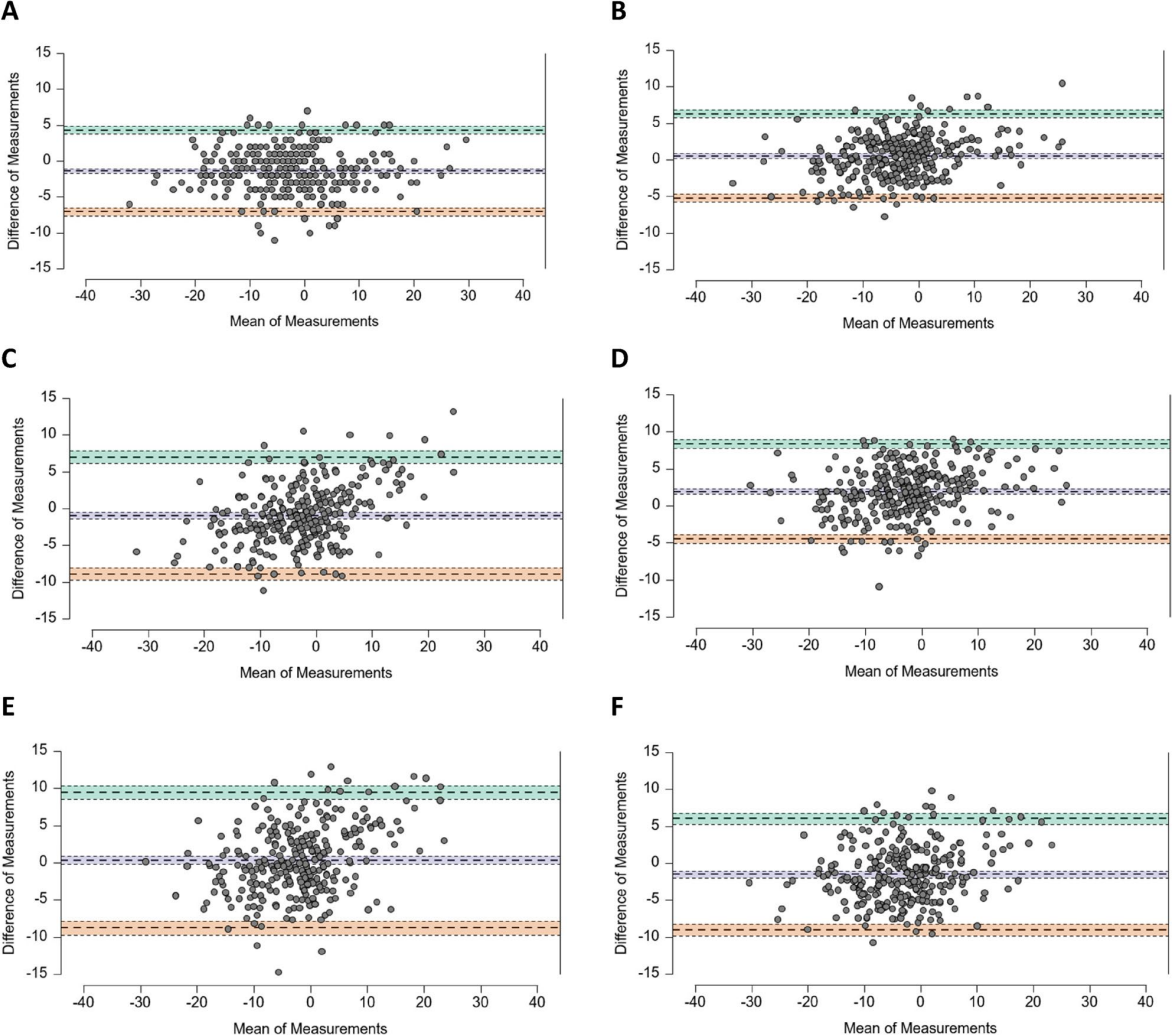
Bland-Altman-Plots with mean difference (Bias) [95% C.I.] and upper and lower limits of agreement (LoA) [95% C.I.] for the agreement between: (**A**) Rater 1 and Rater 2 (1st Read); (**B**) Rater 1 (1st Read) – OpenPose; (**C**) Rater 1 (1st Read) – BlazePose; (**D**) Rater 2 (1st Read) – OpenPose; (**E**) Rater 2 (1st Read) – BlazePose, and (**F**) OpenPose – BlazePose. Data of all knee frontal plane projection angles (FPPA) measured in all functional tests (SLS, SLH and SLL) and Static assessment

A small systematic bias between raters was observed in Bland–Altman analysis (mean difference = − 1.4°, LoA = − 5.8° to 2.9°) (Table 4; Fig. 2A).

**Table 4:** Agreement between raters (app-based application) and AI-based methods

		Mean Difference	Limits of Agreement	
			Lower	Upper
Rater 1 – Rater 2	1st Read	-1.39	-7.09	4.31
	2nd Read	-1.40	-6.48	3.68
Rater 1 (1st Read)	OpenPose	0.53	-5.23	6.28
	BlazePose	-0.96	-8.91	6.99
Rater 2 (1st Read)	OpenPose	1.92	-4.48	8.32
	BlazePose	0.32	-8.78	9.43
OpenPose - BlazePose		-1.50	-9.03	6.03

### Agreement between methods

#### Agreement between application-based and AI-based methods

Following data screening, which led to the exclusion of 31 BlazePose frames (10.8%) due to detection errors, the agreement between application-based and AI-based FPPA measurements was evaluated. The missing data were evenly distributed across participants (χ²(15) = 20.20, *p* = 0.164), suggesting no systematic data quality issues attributable to individual participants.

OpenPose demonstrated the highest agreement with the manual application-based reference. Across all functional tasks and raters, OpenPose reached ICC = 0.94–0.95 and CCC = 0.95 (95% CI 0.94–0.96). The mean bias between OpenPose and manual application-based measurement was 0.53° and 1.92°, for Rater 1 (1st Read) and Rater 2 (1st Read), respectively, indicating slightly higher valgus values from the AI model. The 95% limits of agreement (LoA) ranged from − 5.23° to 6.28° (Rater 1), suggesting narrow dispersion around the mean difference (Table 4; Fig. 2B and D).

BlazePose achieved slightly lower agreement, with ICC = 0.85–0.90 and CCC = 0.89 (95% CI 0.86–0.91). The bias was − 0.96° and 0.32°, for Rater 1 (1st Read) and Rater 2 (1st Read), respectively (Table 4, Fig. 2C and E).

#### Agreement between AI-based methods

The two HPE models showed good but not excellent agreement with each other. The ICC between OpenPose and BlazePose was 0.89 (95% CI 0.87–0.91), and CCC = 0.88 (95% CI 0.86–0.91), with a mean bias of − 1.5° (LoA = − 9.0° to 6.0°) (Table 4; Fig. 1F). This indicates that OpenPose systematically produced slightly higher knee valgus angles than BlazePose.

### Best functional test to evoke functional valgus leg axis deviations

**Fig. 3:**
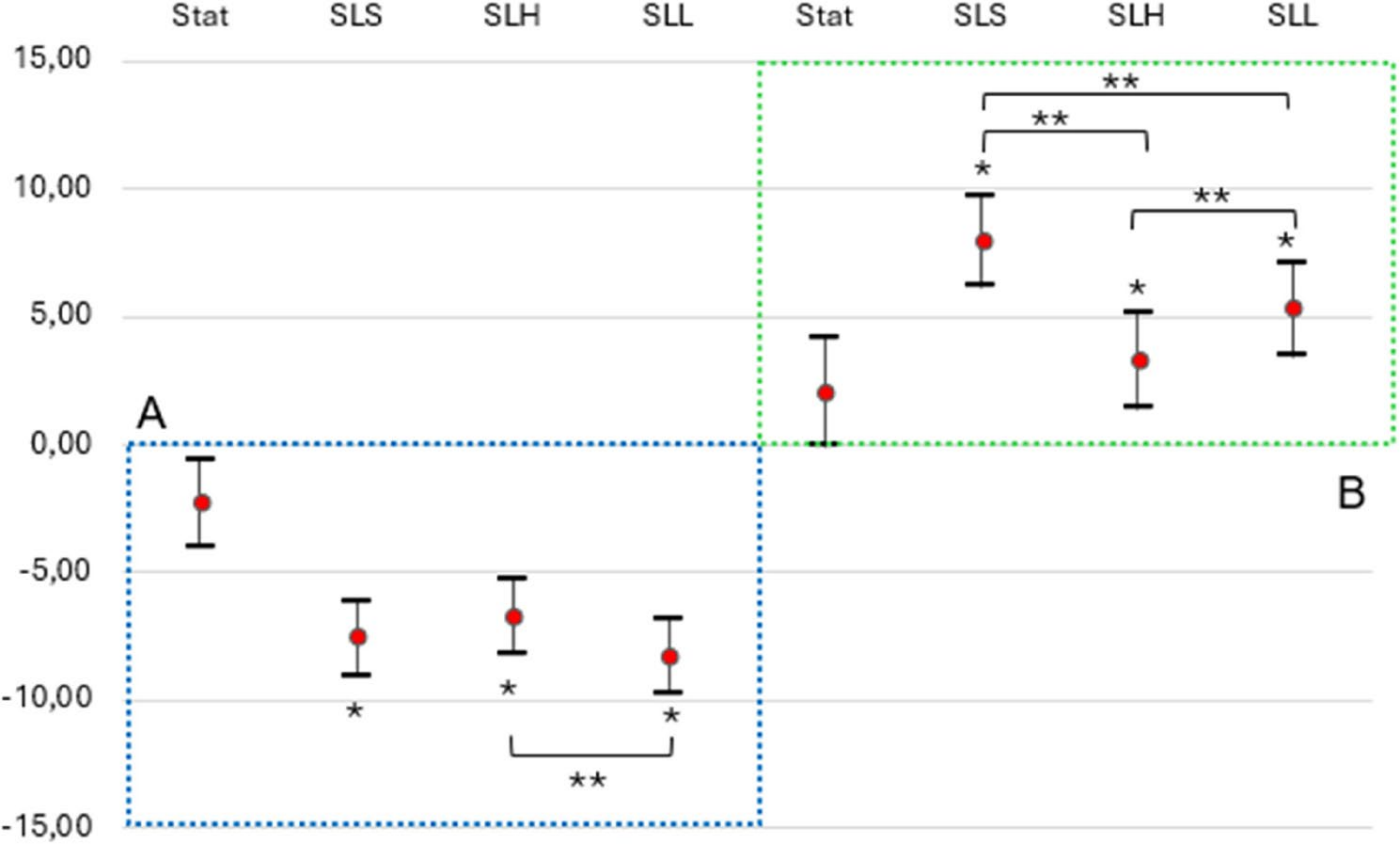
Point estimates (red bullets) and their 95% C.I. for (**A**) LMM model to investigate which functional test is the best to evoke valgus, and (**B**) varus functional leg axis deviations in the frontal plane. * = *P*-values < 0.001, which correspond to test of null hypothesis against 0. ** = *P*-values < 0.01 following Bonferroni adjustment, indicating significant pairwise differences between estimated means. Stat = Static; SLS = Single-Leg Squat; SLH = Single Leg Hop for Distance; SLL = Single Leg Landing

The LMM model showed significant main effects for the fixed effect variable “functional task” (*p* < 0.01) but not for measurement time (*p* = 0.4), rater (*p* = 0.1) and sex (*p* = 0.4). All three functional tasks significantly evoked functional valgus knee angle deviations. The SLL test induced the biggest functional valgus deviations (95% C.I. -9.74 to -6.84) (Table 5 and Fig. 3A). Pairwise comparisons of the estimated marginal means showed a significant contrast between SLL vs. SLH (b= -1.56, SE = 0.47, 95% C.I. [-2.48 to -0.63], *p* < 0.01) and not significant contrasts between SLS vs. SLH (*p* = 0.2) and SLS vs. SLL (*p* = 0.4), indicating that the SLL test was superior to SLH but not superior to SLS in eliciting knee valgus deviations (see Fig. 3).

**Table 5:** Estimated marginal means with 95% C.I. for each functional task in both models

Model	Functional Task	Estimate	SE	95% C.I.		*p*-value*
				Lower	Upper	
Model 1 (valgus)	Static	-2.31	0.88	-4.03	-0.58	< 0.01
	SLS	-7.60	0.75	-9.07	-6.13	< 0.01
	SLH	-6.73	0.74	-8.18	-5.28	< 0.01
	SLL	-8.29	0.74	-9.74	-6.84	< 0.01
Model 2 (varus)	Static	2.02	1.10	-0.14	4.18	0.07
	SLS	7.92	0.91	6.13	9.71	< 0.01
	SLH	3.24	0.95	1.38	5.10	< 0.01
	SLL	5.27	0.94	3.42	7.12	< 0.01

Results are averaged over the levels of: measurement time, Rater and Sex

*SLS* single-leg squat, *SLH* single-leg hop, *SLL* single-leg landing

*= *P*-values correspond to test of null hypothesis against 0

### Best functional test to evoke functional varus leg axis deviations

There were significant main effects for the fixed effect variable “functional task” (*p* < 0.01) and no main effects for measurement time (*p* = 0.4), rater (*p* = 0.05) and sex (*p* = 0.1). Model fit statistics are presented for both models in Table 6. All three functional tasks significantly evoked functional varus knee angle deviations, but the SLS test evoked the biggest varus deviations (95% C.I. 6.13 to 9.71) (Table 5 and Fig. 3B). Pairwise comparisons of the estimated marginal means revealed the largest contrast between SLS vs. SLH (b = 4.68, SE = 0.67, 95% C.I. [3.37 to 5.99], *p* < 0.01). There were also significant contrasts between SLS vs. SLL (b = 2.65, SE = 0.66, 95% C.I. [1.33 to 3.94], *p* < 0.01) and SLL vs. SLH (b = 2.03, SE = 0.69, 95% C.I. [0.68 to 2.95], *p* < 0.01). These results indicate that the SLS test elicited greater functional knee varus deviations than the other two tests (see Fig. 3).

**Table 6:** Fit statistics of both linear mixed models (LMM)

	Number of observations	Deviance (REML)	logLik	AIC	BIC
Model 1	878	5524.00	-2762.00	5542.00	5585.00
Model 2	466	2907.01	-1453.50	2925.01	2962.31

*REML* Restricted maximum likelihood, *logLik* Log-Likelihood, *AIC* Akaike Information Criterion, *BIC* Bayesian Information Criterion

## Discussion

This study investigated the intra- and inter-rater reliability of manual application-based 2D knee FPPA measurements and its agreement with automated AI-based HPE models during three functional single-leg tasks. The findings demonstrate that the manual application-based method using the PhysioMaster^®^ application achieved excellent intra- and inter-rater reliability. Both AI-based models demonstrated good to excellent agreement with manual application-based measurements, with OpenPose outperforming BlazePose. Among the three tasks, the SLL elicited the most pronounced valgus deviations, whereas the SLS most consistently evoked varus alignment.

### Reliability of application-based measurement

The excellent intra- and inter-rater reliability of the manual application-based knee FPPA measurements aligns with prior studies demonstrating high reproducibility of 2D angle measurements in the frontal plane [11, 20]. Such results reinforce that standardized 2D video analysis can provide sufficiently stable data for clinical and research applications, provided that the camera setup, anatomical landmark definition, and evaluator training are consistent.

The lowest intra- and inter-rater reliability estimates were achieved for the knee FPPA measurements in the static position. Five factors might explain these results: (1) the lower sample size for static ICC estimates (only 32 samples; one image per participant per leg) might have introduced a mathematical challenge, since small sample sizes make estimates of between-subject and within-subject variance less stable. Random fluctuations can inflate the within-subject variance relative to the between-subject variance, artificially lowering the ICC; (2) with a smaller sample size, the confidence intervals around the ICC widen, often including values near zero, which reduces the apparent reliability; (3) in the static position, valgus and varus deviations are very small, making it more difficult to accurately place the vector endpoints over the anatomical landmarks; (4) The between-subject variability in the static measurements was substantially smaller than in the functional tests, yielding a narrower total variance against which measurement error is evaluated in the ICC; and (5) the app recorded angles as integers only, imposing a fixed error floor of 1°. Given the narrow variance in the static condition, this rounding error constituted a proportionally larger share of total variance, thereby attenuating the corresponding ICC values.

### Agreement between AI-based and manual application-based methods

OpenPose demonstrated excellent agreement with manual application-based measurements, whereas BlazePose showed good but less consistent agreement, reflected by wider limits of agreement and a tendency toward underestimating valgus alignment. These findings align with previous work showing that OpenPose achieves high accuracy in frontal-plane kinematics [24, 25], while BlazePose exhibits greater variance in lower-limb detection [27, 28]. A likely explanation for the observed differences in agreement lies in the underlying architectural design of the two HPE models. OpenPose follows a bottom-up approach, in which all body landmarks are detected simultaneously across the image before being assigned to an individual skeleton [22]. This strategy allows robust landmark detection even when parts of the body are partially occluded or outside the field of view, as spatial relationships between keypoints are inferred globally rather than sequentially. In contrast, BlazePose is based on a top-down architecture that first detects a person-level bounding box and subsequently estimates joint locations within this predefined region [21]. While this approach enables efficient real-time performance and low computational cost, it relies strongly on accurate full-body detection. Consequently, incomplete body visibility or truncated image regions may compromise downstream joint localization. In the present study, facial regions were intentionally cropped from all images to ensure anonymization. This preprocessing step likely affected BlazePose more strongly than OpenPose, as head and upper-body landmarks contribute to stable person detection and pose normalization in top-down pipelines. The increased number of missing or implausible landmark detections observed for BlazePose supports this assumption. These architectural characteristics may explain the wider limits of agreement and the tendency toward valgus underestimation observed for BlazePose. Similar sensitivities of top-down HPE models to occlusions and reduced field-of-view conditions have been reported previously [23, 27].

### Best functional task to evoke valgus or varus knee angle deviations

The LMM findings confirmed that task selection substantially influenced the measured knee FPPA values. SLL elicited the most pronounced valgus deviations, consistent with literature that shows increased medial knee collapse during landing tasks [35, 36]. SLS, in contrast, produced the strongest varus knee angles, likely reflecting lateral loading strategies and hip–knee control patterns during slow, controlled descent. These results illustrate that the choice of the functional test can shape the type and magnitude of leg axis deviation observed. For valgus-sensitive screening (e.g., ACL risk profiling), landing tasks may be preferable, while SLS may be suitable when assessing control strategies associated with -varus movement patterns.

#### Clinical implications

For physiotherapists and sports scientists, the present results suggest that both, application-based and AI-based 2D analyses can be used to assess knee valgus and varus angles reliably within the scope of functional testing. OpenPose, in particular, may serve as a time-efficient tool for semi-automated screening or follow-up assessments, where marker-based systems are impractical, like in the context of a physiotherapy examination within a treatment session. BlazePose, though slightly less precise, offers advantages in portability and processing speed and can be implemented on common mobile devices, which may facilitate future tele-rehabilitation or home-based monitoring applications.

Previous intervention studies reported that changes in dynamic knee valgus following neuromuscular or strength-based training are typically modest and often lie within a range of approximately 5–10 degrees [6, 7, 37]. In the present study, the limits of agreement of the applied AI-based methods were of a comparable magnitude, indicating that small training-related changes may fall within the measurement variability. Consequently, it remains unclear whether such differences reflect true neuromuscular and biomechanical adaptations or measurement errors, particularly at the individual level. Repeated measurements may improve interpretability by reducing random measurement errors, for example by averaging multiple trials per task and time point. However, repeated measurements cannot eliminate systematic model-specific bias and may be limited by natural within-subject movement variability. Therefore, AI-based angle estimation should complement, not replace, clinical expertise, particularly when assessing subtle improvements or interventional effects.

### Limitations & strengths

Several limitations should be considered when interpreting the present findings. First, the study sample was relatively small and consisted exclusively of healthy adults, which limits generalizability to clinical populations or individuals with musculoskeletal impairments. Second, the analysis was restricted to 2D knee FPPA derived from single still images. While this approach reflects common clinical documentation practices, it does not capture temporal movement characteristics, within-trial variability, or three-dimensional joint mechanics, which may be particularly relevant during dynamic tasks such as single-leg landings [4]. Furthermore, the selected frame corresponded to the moment when the hip joint reached its lowest vertical position, however, frontal-plane hip displacement may not coincide with peak knee valgus/varus angles. Participant-related visual characteristics, including skin tone, clothing contrast, and lighting conditions, may have influenced landmark detection accuracy in the AI-based models as well as the application-based method. As many pose estimation algorithms are trained on datasets with limited demographic diversity, model performance may vary across populations, potentially affecting generalizability. In addition, facial regions were cropped to ensure anonymization (Fig. 1A-C), which affected the top-down pose estimation model OpenPose, which relies on full-body context for stable joint localization. Manual application-based measurements were performed by trained raters (LJ and LMM) under standardized conditions. Although excellent reliability was observed, measurement consistency may differ when assessments are conducted by clinicians with varying levels of experience or training. Furthermore, no marker-based three-dimensional motion capture system was used as an external reference, meaning that agreement was evaluated relative to manual application-based 2D assessment rather than absolute biomechanical ground truth. Finally, the findings are inherently dependent on the specific pose estimation models evaluated. While OpenPose and BlazePose are well-established and widely used frameworks, OpenPose in particular represents an earlier generation of human pose estimation architectures. Recent advances in pose estimation, including whole-body and real-time frameworks such as AlphaPose [38] and YOLO-Pose [39], have demonstrated improved robustness and accuracy under challenging conditions. Therefore, the present results should be interpreted as model-specific and may not directly translate to newer pose estimation approaches without further empirical validation.

A key strength of this study is the direct comparison of manual application-based and AI-based 2D knee angle assessment using the same dataset, enabling method comparison under identical conditions. The inclusion of multiple functional single-leg tasks allowed task-specific interpretation of knee alignment. In addition, the combined use of reliability, agreement, and linear mixed model analyses provided a comprehensive evaluation of measurement consistency and task-related effects. The clinically feasible setup further supports the relevance of the findings for physiotherapy practice.

Future research should investigate AI-based pose estimation in larger and more diverse populations, including individuals with musculoskeletal impairments. Dynamic, frame-by-frame analyses across complete motion sequences could improve the sensitivity of detecting compensatory strategies and temporal asymmetries. Integrating 2D and 3D pose estimation, or combining AI-based methods with wearable sensors, may further enhance accuracy and clinical applicability. Moreover, open benchmarking studies comparing multiple HPE frameworks under standardized conditions would be valuable for establishing reference metrics in physiotherapy research.

## Conclusion

This study demonstrated that both, application-based and AI-based two-dimensional (2D) approaches, can be used to assess frontal-plane knee alignment during functional single-leg tasks, albeit with different methodological strengths. The manual application-based approach (PhysioMaster^®^ application) achieved excellent intra- and inter-rater reliability, confirming its suitability for repeated clinical assessments. The AI-based model OpenPose demonstrated excellent agreement with manual application-based measurements, whereas BlazePose showed good agreement with greater measurement variability. Further research comparing the planar angles reported here with three-dimensional motion capture measurements is warranted to assess the concurrent validity of those methods against the golden standard.

Across functional tasks, knee alignment patterns differed systematically. Single-Leg Landing elicited the most pronounced valgus deviations, suggesting its suitability for identifying valgus-dominant movement strategies and potential knee collapse tendencies. In contrast, the Single-Leg Squat consistently produced varus-oriented alignment, indicating higher demands on lateral knee and hip control during slow, controlled movement.

Overall, AI-assisted movement analysis appears feasible and time-efficient for use in physiotherapy settings. However, because typical training-related changes in knee valgus lie within the range of measurement variability, individual-level changes should be interpreted with caution. AI-based angle estimation should therefore complement, rather than replace, clinical reasoning, particularly when subtle intervention effects are evaluated.

## Supplementary Information

Supplementary Material 1. Appendix Table A1.

## Data Availability

All images used for data extraction, the raw data, the code and the outputs of all statistical analyses are not available publicly for data protection reasons, but are available from the corresponding authors on reasonable request.

## Abbreviations

AI: Artificial Intelligence
DKV: Dynamic Knee Valgus
HPE: Human Pose Estimation
SLS: Single-Leg Squat
SLH: Single-Leg Hop for Distance
SLL: Single-Leg Landing
CCC: Lin’s Concordance Correlation Coefficient
ICC: Intraclass Correlation Coefficients
LMM: Linear Mixed Model
FPPA: Frontal Plane Projection Angle
STROBE: Strengthening the Reporting of Observational Studies in Epidemiology
GRRAS: Guideline for Reporting Reliability and Agreement Studies
2D: Two-Dimensional
3D: Three-Dimensional
