# Supplementary material for: Assessment of dynamic knee angle deviations in the frontal plane in physiotherapy clinical practice: intra- and inter-rater reliability of an application and agreement with two AI-models
Source: BMC Musculoskelet Disord. 2026 May 12;27:407. doi: 10.1186/s12891-026-09958-9 (PMC13162436; doi:10.1186/s12891-026-09958-9)
Supplement: Supplementary file 1 — Supplementary Material 1. Appendix Table A1. [file 12891_2026_9958_MOESM1_ESM.docx]

Appendix Table

**Table A1** – Description of the standardized instructions used during functional testing and criteria for invalid attempts

| **Test** | **Standardized Instructions** | **Attempt Invalid** |
| --- | --- | --- |
| Single-Leg Squat  (SLS) | 1. Stand on the leg to be tested. Keep the other leg in the air in front of the body without touching the floor. 2. Hold your arms stretched forward, parallel to the floor. 3. Bend the knee slowly, as far as possible, while maintaining balance. 4. Keep the upper body as upright as possible. 5. Return controlled to the starting position. | - Loss of balance - Readjusting the tested foot on the floor - The non-tested (free) leg touches the floor |
| Single-Leg Hop for Distance  (SLH) | 1. Stand on the leg to be tested with a slightly bent knee. 2. Jump forward as far as possible. 3. Land on the same leg and hold your balance for at least 3 seconds. 4. The jumping leg must be the only leg that touches the ground during the entire test. | - Balance cannot be held for 3 seconds - Readjusting the tested foot after landing - The non-test (free) leg touches the ground |
| Single-Leg Landing  (SLL) | 1. Stand on the bench, on the leg you are testing. 2. Jump forward off the bench with your test leg, and land on that same leg. 3. Try to hold your balance for at least 3 seconds after landing. 4. The jumping leg must be the only leg that touches the ground during the entire test. | - Cannot maintain balance for 3 seconds - Readjusting the tested foot after landing - The non-test (free) leg touches the ground |
